# (*Z*)-5-(2,4-Dihydroxybenzylidene)thiazolidine-2,4-dione Prevents UVB-Induced Melanogenesis and Wrinkle Formation through Suppressing Oxidative Stress in HRM-2 Hairless Mice

**DOI:** 10.1155/2016/2761463

**Published:** 2016-05-08

**Authors:** Bonggi Lee, Kyoung Mi Moon, Seong Jin Kim, So Hee Kim, Dae Hyun Kim, Hye Jin An, Ji Won Jeong, Ye Ra Kim, Sujin Son, Min Jo Kim, Ki Wung Chung, Eun Kyeong Lee, Pusoon Chun, Young Mi Ha, Min-Sun Kim, Sang Hyun Mo, Hyung Ryong Moon, Hae Young Chung

**Affiliations:** ^1^College of Pharmacy, Pusan National University, Busan 609-735, Republic of Korea; ^2^Molecular Inflammation Research Center for Ageing Intervention (MRCA), Pusan National University, Republic of Korea; ^3^College of Pharmacy, Inje University, Busan 609-735, Republic of Korea; ^4^Department of Chemistry, Dong-A University, Busan 609-735, Republic of Korea; ^5^College of Pharmacy, Sunchon National University, Sunchon, Republic of Korea; ^6^Bio-FD&C, Songdo Mirae-ro 30, Incheon, Republic of Korea

## Abstract

*Background*. Uncontrolled melanogenesis and wrinkle formation are an indication of photoaging. Our previous studies demonstrated that (*Z*)-5-(2,4-dihydroxybenzylidene)thiazolidine-2,4-dione (MHY498) inhibited tyrosinase activity and melanogenesis* in vitro*.* Objective*. To examine* in vivo* effects of MHY498 as an antiaging compound on UVB-induced melanogenesis and wrinkle formation, we topically applied MHY498 on dorsal skin of HRM-2 hairless mice.* Methods*. Using histological analysis, we evaluated effects of MHY498 on melanogenesis and wrinkle formation after UVB exposure. In addition, related molecular signaling pathways were examined using western blotting, fluorometric assay, and enzyme-linked immunosorbent assay.* Results*. MHY498 suppressed UVB-induced melanogenesis by inhibiting phosphorylation of CREB and translocation of MITF protein into the nucleus, which are key factors for tyrosinase expression. Consistently, tyrosinase protein levels were notably reduced in the dorsal skin of the hairless mice by MHY498 treatment. Furthermore, MHY498 inhibited UVB-induced wrinkle formation and collagen fiber destruction by increasing type 1 procollagen concentration and decreasing protein expression levels of MMPs, which play an essential role in collagen fiber degradation. As a mechanism, MHY498 notably ameliorated UVB-induced oxidative stress and NF-*κ*B activation in the dermal skin of the hairless mice.* Conclusion*. Our study suggests that MHY498 can be used as a therapeutic or cosmetic agent for preventing uncontrolled melanogenesis and wrinkle formation.

## 1. Introduction

Skin plays an important role as protective barrier against ultraviolet (UV) irradiation and environmental pollution. Because human skin is continuously exposed to various environmental factors, skin aging may be an inevitable natural process. However, preventing UV-induced skin ageing is important because short-term UV exposure can cause sunburn, inflammation, immune suppression, and dermal connective tissue damage [1] and chronic exposure to UV can ultimately cause photoaging and skin cancer through increasing oxidative stress [2, 3]. Uncontrolled melanogenesis and collagen fiber destruction followed by wrinkle formation are a hallmark of photoaging. Wrinkle formation is closely associated with collagen fibrils [1]. It is characterized by decreased skin elasticity and degeneration of the extracellular matrix (ECM) such as collagen which is produced by fibroblasts in the dermis [4]. Therefore, preventing collagen fiber destruction is essential for inhibiting wrinkle formation.

On the other hand, melanogenesis is a protective process of skin against UV-induced damage [5], but chronic and repeated exposures to UV can lead to premature aging of the skin [5]. Furthermore, melanin accumulation is directly associated with pigmentation disorders such as melasma, freckles, and solar lentigo [6, 7]. Melanocyte is located in the basal layer of the epidermis and a main cell type to secrete melanin. Tyrosinase is an important enzyme responsible for skin pigmentation in mammals [8], which catalyses two rate-limiting steps in melanogenesis: the hydroxylation of tyrosine to 3,4-dihydroxyphenylalanine (DOPA) and the oxidation of DOPA to DOPAquinone. Therefore, tyrosinase plays an essential role in melanin production in melanocytes and inhibiting tyrosinase is an attractive target for ameliorating pigmentation disorders and various cosmetics.

Oxidative stress plays a synergistic role in UV-induced skin damage. Reactive oxygen species (ROS) such as ∙O_2_^−^, ∙OH, and NO can be induced by UV and the accumulated ROS eventually causes wrinkle formation and melanogenesis in skin [9]. As a mechanism, ROS activate NF-*κ*B signaling, which is responsible for inflammation in skin [10]. In addition, it has been shown that ROS and NF-*κ*B activation stimulates expression and activation of matrix metalloproteinases (MMPs) that stimulate collagen degradation and inhibit collagen synthesis [9, 11]. Therefore, preventing ROS generation and thereby reducing oxidative stress are important targets for inhibiting UV-induced skin damage.

Our previous* in vitro* study reported that (*Z*)-5-(2,4-dihydroxybenzylidene)thiazolidine-2,4-dione (MHY498) suppressed melanogenesis by directly inhibiting tyrosinase [12] and interfering with tyrosinase expression [13]. However,* in vivo* effects of MHY498 on skin pigmentation and wrinkle formation have not been examined. Although various compounds for inhibiting pigmentation or wrinkle formation have been screened in cell models, they are not effective in animal models, possibly due to low stability and low selectivity. Here, our current study examined* in vivo* effects of MHY498 on UVB-mediated skin aging including melanogenesis and wrinkle formation using HRM-2 hairless mice. The data showed that MHY498 notably reduced UVB-induced pigmentation, collagen fiber destruction, and wrinkle formation in the dorsal skin of hairless mice at least partially through reducing UVB-induced oxidative stress and related signaling.

## 2. Materials and Methods

### 2.1. Mice

HRM-2 hairless mice (6-week-old males) were obtained from Hoshino Laboratory Animals (Yashio, Saitama, Japan). The mice were maintained with 12 h/12 h light/dark cycle and received ad libitum access to standard laboratory diet and water. MHY498 (200 *μ*L of 0.5 *μ*M and 5 *μ*M) was prepared in a solution containing propylene glycol and ethanol (3 : 7). MHY498 solution or the sham solution including propylene glycol and ethanol was topically applied to a designated site (3 cm  ×  3 cm) on dorsal skin of hairless mice daily over the whole study period. Specifically, MHY498 or the sham solution was pretreated for 3 days before UVB (302 nm) exposure. From day 4 to day 25, 2 h after MHY498 or the sham solution treatment, mice were exposed to UVB every other day for 1 h in an UVB exposure chamber at 150 mJ/cm^2^ (see Table 1 for treatment scheme). From day 26, UVB was exposed every day to maximize the effect of UVB on skin pigmentation and wrinkle formation based on our preliminary experiments to pick up the best UVB treatment condition (Table 1). After 28 days of the treatments, the mice were sacrificed and dorsal skin was excised and quickly frozen in a nitrogen tank for western blotting and measuring oxidative stress. For staining purpose, the excised skin was fixed in 4% paraformaldehyde. All animal studies were approved by the Institutional Animal Care Committee of Pusan National University and were performed in accordance with the guidelines for animal experiments issued by Pusan National University.

### 2.2. UVB Exposure

We used a commercially available UV exposure chamber (Ultraviolet Crosslinker, UVP, LLC, Upland, CA, USA) for chronic UVB exposure of mice [14, 15]. The UV exposure chamber is designed to measure and control the ultraviolet (UV) radiation within the exposure chamber. We used UVB specific tube and sensor provided by the company that continually measures the UVB energy and automatically adjusts to variations in UVB intensity that occur as the UVB tubes age. Therefore, we could minimize potential contamination of UVA, UVC, and infrared radiation.

### 2.3. Evaluation of Depigmenting Activities* In Vivo*

After treatment of MHY498 followed by UVB exposure, darkness of skin sites was measured on day 28 of the treatment using a CR-10 spectrophotometer (Konica Minolta Sensing, Inc., Sakai, Osaka, Japan), which describes colors using *L* ^*∗*^ (higher and lower values mean whiter and blacker, resp.), as described by the Commission Internationale de l'Eclairage color system. When we compared nontreated mice with the control mice treated with the sham solution containing propylene glycol and ethanol, there was no difference in skin brightness values after UVB exposure. Therefore, we only used mice treated with the sham solution as a control group.

### 2.4. Fontana-Masson Staining

Fontana-Masson staining was performed to detect melanin formation in skin of hairless mice. Fresh skin samples were fixed in 4% paraformaldehyde overnight at room temperature and stained for detecting melanin using a Fontana-Masson staining kit (American Mastertech, Inc., Lodi, CA, USA). Briefly, sliced skin samples were stained with ammoniacal silver solution for 60 min at 60°C. The samples were incubated in 0.1% gold chloride followed by 5% sodium thiosulfate. Melanin spots were observed using an AE-31 light microscopy (Motic, Hong Kong).

### 2.5. Masson's Trichrome Staining

Masson's trichrome staining was performed as previously described [16]. Fresh skin samples were fixed in 4% paraformaldehyde overnight at room temperature and paraffin-embedded skin specimens were sectioned at 5 *μ*m, deparaffinized, and stained with Masson's trichrome to visualize collagen fibers. Staining tissue sections were examined under an optical microscope (Eclipse TS100; Nikon Instruments Inc., Melville, NY, USA).

### 2.6. Western Blotting

Protein samples from skin lysates (30 *μ*g) were separated by sodium dodecyl sulfate-polyacrylamide gel and transferred to polyvinylidene fluoride (PVDF) membranes, which were immediately placed in 5% nonfat milk blocking buffer containing 10 mM Tris (pH 7.5), 100 mM NaCl, and 0.1% Tween 20. The membrane was washed in TBS-Tween buffer for 30 min and then incubated with specific primary antibodies indicated in the figure legends (dilution 1 : 1000) at 4°C overnight. After washing with TBS-Tween buffer, the membrane was incubated with a horseradish peroxidase-conjugated anti-mouse antibody (Santa Cruz, 1 : 10,000), an anti-rabbit antibody (Santa Cruz, 1 : 10,000), or an anti-goat antibody (Santa Cruz, 1 : 10,000) at 25°C for 1 h. The immunoblots were visualized using Western Bright Peroxide solution (Advansta, CA, USA) and Davinch-Chemi CAS-400 (Davinch-K, Korea) according to the manufacturer's instructions. Antibodies used in this study are the following: p-CREB (SC-101663), tyrosinase (SC-15341), MITF (SC-11002), MMP1 (SC-12348), MMP9 (SC-6840), MMP12 (SC-30072), MMP13 (SC-12363), type 1 procollagen (SC-25973), type 3 procollagen (SC-8779), p-p65 (S536) (SC-33020), iNOS (SC-8310), *β*-actin (SC-47778), and TFIIB (SC-225).

### 2.7. Measurement of ROS

ROS generation was measured as previously described [17]. Based on the oxidation of nonfluorescent 2′,7′-dichlorofluorescein diacetate (DCF-DA) to highly fluorescent 2′,7′-dichlorofluorescein (DCF) in the presence of esterases and ROS, including lipid peroxides, a fluorometric assay was used to examine ROS levels in the dorsal skin of hairless mice. Briefly, DCF-DA (50 *μ*M) was mixed with skin homogenates (10 *μ*L) to a final volume of 250 *μ*L. Fluorescence intensity was recorded every 5 min for 30 min using a fluorescence plate reader (GENios, Tecan Instruments, Salzburg, Austria) at emission and excitation wavelengths of 530 and 485 nm, respectively.

### 2.8. Measurement of ONOO^−^

Peroxynitrite (ONOO^−^) generation was measured as previously described [17]. Peroxynitrite (ONOO^−^) generation was examined by measuring the oxidation of DHR-123. Briefly, dorsal skin homogenate (10 *μ*L) was mixed with the rhodamine solution (50 mM sodium phosphate buffer, 90 mM sodium chloride, 5 mM diethylenetriaminepentaacetate [DTPA], and DHR-123). Changes in fluorescence intensity were observed every 5 min for 30 min using a fluorescence plate reader, with excitation and emission wavelengths at 485 and 535 nm, respectively.

### 2.9. Histological Analysis of Skin

We performed histological analysis of the dorsal skin using a commercially available silicone impression material (SILFLO) to mold the wrinkle on dorsal skin in silicone. We followed the manufacturer's instruction for preparing skin samples. We used a commercially available institution to get images of wrinkle using the molded skin samples (Oriental Medicine Industry Support Center, South Korea).

### 2.10. Statistical Analysis

All results are expressed as mean ± SEM. Treatments were compared using one-way ANOVA followed by Bonferroni test. *P* values <0.05 were considered statistically significant.

## 3. Results and Discussion

### 3.1. Effect of MHY498 on UV-Induced Skin Pigmentation of HRM-2 Hairless Mice

**Figure 1:**
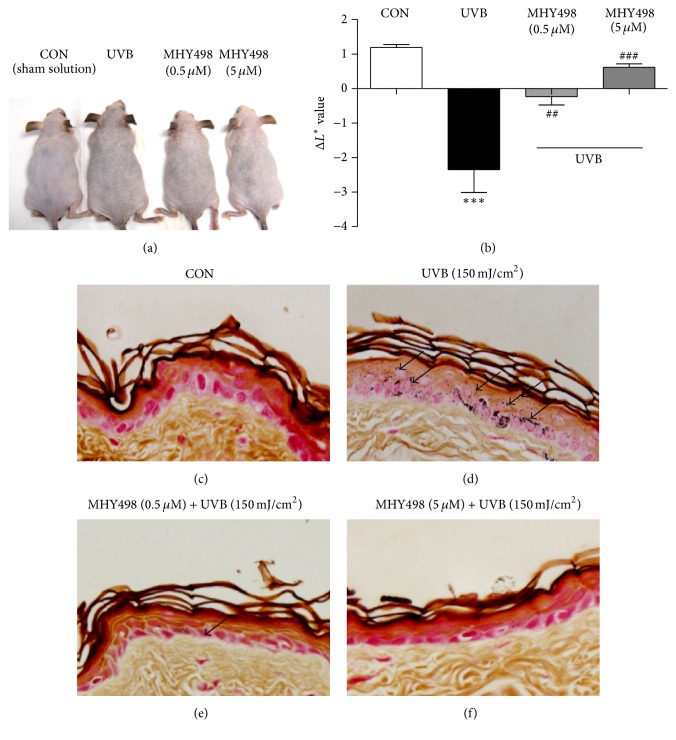
MHY498 ameliorates UVB-induced skin pigmentation. MHY498 was pretreated for 3 days. From day 4, 2 h after MHY498 application to the dorsal skin of the hairless mice, UVB was exposed to the mice as shown in Table 1 (*n* = 11/group). After 4 weeks, pictures of the dorsal skin were taken (a). Darkness of the skin sites was measured on day 28 of the treatment using a CR-10 spectrophotometer (Konica Minolta Sensing, Inc., Sakai, Osaka, Japan). Higher *L* ^*∗*^ values represent whiter colors (*n* = 11/group) (b). After MHY498 application followed by UVB exposure, skin samples were excised and Fontana-Masson staining was performed as described in Materials and Methods (c–f). Microscopic analysis of dorsal skin sections from (c) control mice without UVB exposure, (d) mice with UVB exposure, and (e) MHY498 (0.5 *μ*M) or (f) MHY498 (5 *μ*M) treated mice. Arrows represent where melanogenesis was induced by UVB. Data are represented as mean ± SEM. ^##^ *P* < 0.01 and ^###^ *P* < 0.001 compared to a control group without UVB exposure ^*∗∗∗*^ *P* < 0.001 compared to control group with UVB exposure.

We first examined whether MHY498 has cytotoxic effects on Hs27 human dermal fibroblasts and B16F10 mouse skin melanoma cells. Data showed that MHY498 has no cytotoxic effects on both cell lines up to 10 *μ*m (Supplementary Figures  1(a) and 1(b) in Supplementary Material available online at http://dx.doi.org/10.1155/2016/2761463). Also, when we treated the sham or MHY498 solution on dorsal skin of hairless mice for 28 days, no visible evidence of skin irritation was found (Figure 1(a)). To examine* in vivo* effects of MHY498 on skin pigmentation, we topically applied the sham or MHY498 solution to the dorsal skin of the hairless mice for 3 days. From day 4, UVB was exposed to the skin 2 h after MHY498 treatment. Repeated UVB exposure (150 mJ/cm^2^) for 4 weeks darkened skin of mice as expected (Figure 1(a)). However, MHY498 treatment at 0.5 *μ*M or 5 *μ*M markedly ameliorated the UVB-induced darkening of the skin (Figure 1(a)). To confirm the brightening effect of MHY498 on the skin, we measured darkness of the skin using CR-10 spectrophotometer. UVB exposure reduced *L* ^*∗*^ values in which higher values represent whiter color (Figure 1(b)). However, MHY498 treatment recovered UVB-induced pigmentation of the skin (Figure 1(b)). To investigate whether the MHY498-mediated brightening effect of the skin is due to inhibition of melanogenesis, we performed Fontana-Masson staining of the dorsal skin sections. Compared to the control group (Figure 1(c)), UVB exposure induced melanogenesis evidenced by black spots of epidermis (Figure 1(d)). However, MHY498 treatment at 0.5 *μ*M was enough to reduce melanogenesis (Figure 1(e)) and MHY498 at 5 *μ*M fully recovered UVB-induced melanogenesis (Figure 1(f)). These data suggest that MHY498 ameliorates UVB-induced skin darkening in mice, probably through inhibiting melanogenesis.

### 3.2. Effect of MHY498 on UV-Induced Wrinkle Formation and Collagen Fiber Destruction of HRM-2 Hairless Mice

**Figure 2:**
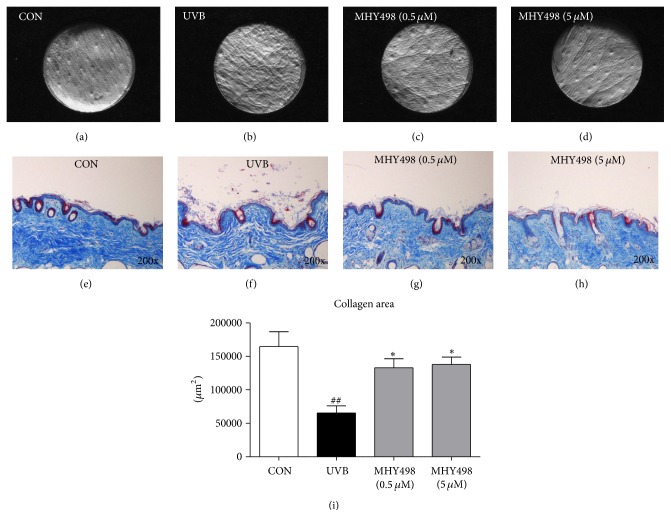
MHY498 ameliorates UVB-induced collagen fiber destruction. MHY498 was pretreated for 3 days. From day 4, 2 h after MHY498 application to the dorsal skin of the hairless mice, UVB was exposed to the mice every other day for 1 h. After 4 weeks, skin samples were excised and Masson's trichrome staining was performed as described in Materials and Methods (a–d). Control mice without UVB exposure (a), mice with UVB exposure (b), and MHY498 (0.5 *μ*M) (c) or MHY498 (5 *μ*M) treated mice (d). Collagen staining appears blue. Collagen area was calculated based on microscopic analysis of the dorsal skin staining (*n* = 11/group) (e). Data are represented as mean ± SEM. ^##^ *P* < 0.01 compared to a control group without UVB exposure and ^*∗*^ *P* < 0.05 compared to the group with UVB exposure.

We examined whether MHY498 has an inhibitory effect on UV-mediated wrinkle formation by histological analysis of the dorsal skin samples. UVB exposure visibly increased wrinkle formation compared to the control group, whereas MHY498 treatment markedly reduced it (Figures 2(a)–2(d)). Because wrinkle formation is closely associated with impairment of skin structure and collagen fiber, we performed Masson's trichrome staining of the dorsal skin sections. Compared to the control group showing dense collagen fiber structure (Figure 2(e)), UVB exposure induced collagen fiber destruction with hyperkeratosis, a hallmark of chronic UV exposure (Figure 2(f)). However, MHY498 treatment markedly decreased these features (Figures 2(g)-2(h)). Consistently, reduced collagen area after UVB exposure was recovered by MHY498 treatment (Figure 2(i)). These data indicate that inhibiting collagen fiber destruction and hyperkeratosis may contribute to the antiwrinkle effect of MHY498 after UVB exposure.

### 3.3. MHY498 Inhibits UVB-Induced CREB Phosphorylation and MITF Translocation into the Nucleus

**Figure 3:**
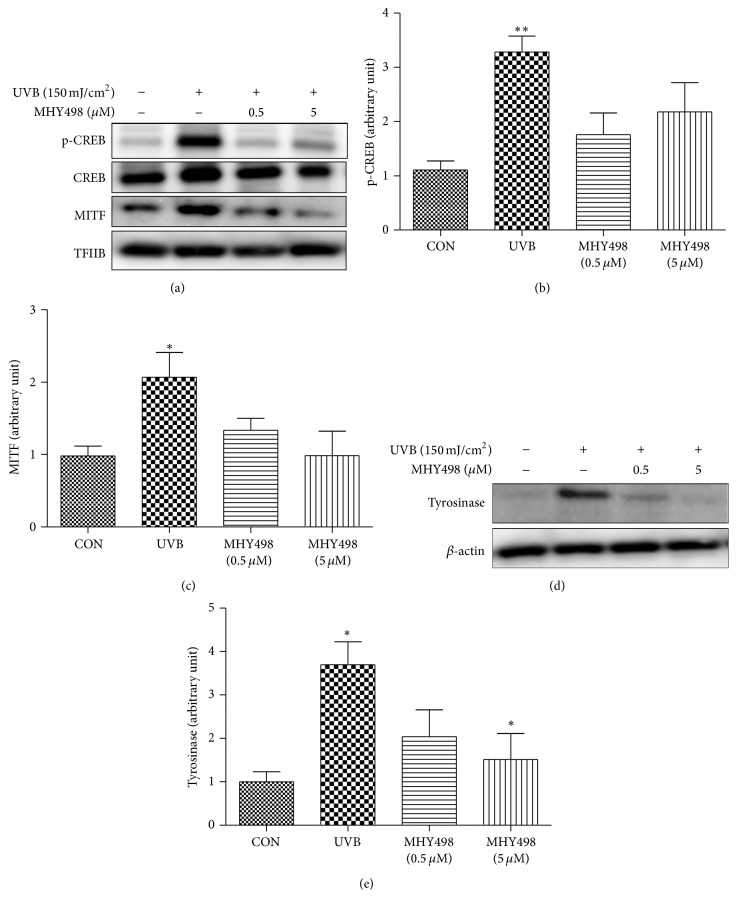
MHY498 reduces CREB phosphorylation and MITF translocation into nucleus. MHY498 was pretreated for 3 days. From day 4, 2 h after MHY498 application to the dorsal skin of the hairless mice, UVB was exposed to the mice every other day for 1 h. After 4 weeks, skin samples were excised and homogenated. Western blotting was performed using skin tissues for detecting (a) p-CREB, CREB, MITF, and TFIIB in nucleus fraction and (d) tyrosinase and *β*-actin in cytosol fraction (*n* = 4/group). The protein levels of (b) p-CREB, (c) MITF, and (e) tyrosinase were semiquantified using Image J software. p-CREB and MITF protein levels were normalized by TFIIB (*n* = 4/group). Tyrosinase protein levels were normalized by *β*-actin. A representative blot is shown from four experiments that yielded similar results. Data are represented as mean ± SEM. ^*∗*^ *P* < 0.05 and ^*∗∗*^ *P* < 0.01 compared to control group without UVB exposure.

Our previous study reported that MHY498 inhibits nitric oxide-induced tyrosinase expression* in vitro *[13]. To examine molecular pathway(s) underlying the antimelanogenic effect of MHY498 on the skin of mice, we investigated protein levels of key molecules that play an important role in melanogenesis. Adenosine 3′,5′-cyclic monophosphate (cAMP) response element binding protein (CREB) and microphthalmia transcription factor (MITF), which induce transcription of tyrosinase, are key transcription factors for melanogenesis [18]. Our data showed that UVB exposure markedly increased phosphorylated CREB, an active form of CREB, in the nucleus, but MHY498 treatment reduced phosphorylated CREB to the level comparable to the control group without UVB exposure (Figures 3(a) and 3(b)). Furthermore, increased MITF translocation into the nucleus by UVB exposure was significantly decreased by MHY498 treatment (Figures 3(a) and 3(c)). These data suggest that MHY498 efficiently blocks CREB activation and MITF translocation into the nucleus. In parallel with this, UVB-induced increase in the protein level of tyrosinase was notably reduced by MHY498 treatment (Figures 3(d) and 3(e)). Our previous* in vitro* study showed that MHY498 scavenges NO, suppresses a NO-mediated signaling pathway, and thus subsequently downregulated tyrosinase expression and melanogenesis in B16F10 melanoma cells [13]. The current study confirmed that MHY498 is effective in preventing UVB-induced melanogenesis* in vivo.*

It has been reported that melanogenesis may be a protective mechanism against UV-induced DNA damage [19, 20]. To examine whether the MHY498-mediated inhibition of melanogenesis increases DNA damage, we measured protein levels of DNA damage markers such as phospho-P53, ATR, and phosphohistone H2AX using the nucleus fraction of skin homogenate. As expected, UVB exposure increased protein levels of phospho-P53 (Ser15) and phosphohistone H2AX (Ser139), whereas MHY498 treatment appears to reverse them (Supplementary Figure  2). There was no clear change of ATR protein levels in response to UVB or MHY498 (Supplementary Figure  2). These data indicate that MHY498-mediated inhibition of melanogenesis may not increase DNA damage in skin.

### 3.4. MHY498 Inhibits UVB-Induced MMP Protein Expression

**Figure 4:**
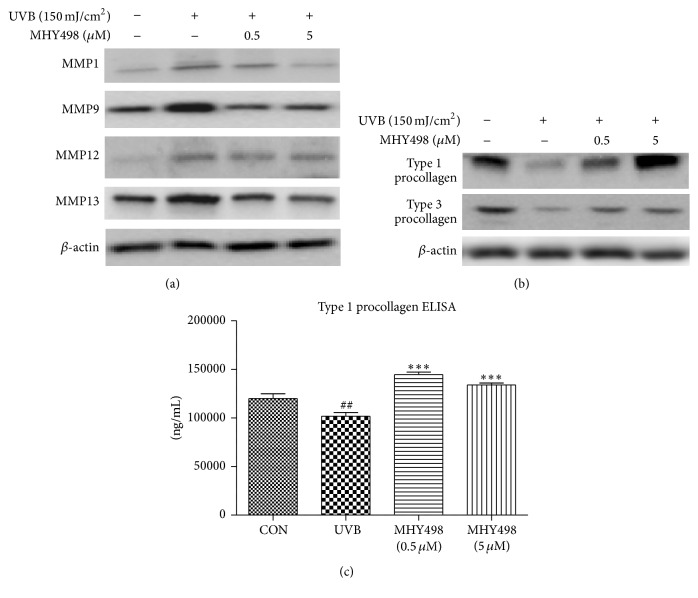
MHY498 reduces protein levels of MMPs and procollagens. MHY498 was pretreated for 3 days. From day 4, 2 h after MHY498 application to the dorsal skin of the hairless mice, UVB was exposed to the mice every other day for 1 h. After 4 weeks, skin samples were excised and homogenated. Western blotting was performed to detect protein levels of MMP1, MMP9, MMP12, MMP13, and *β*-actin (*n* = 4/group) (a). Protein levels of type 1 and type 3 procollagen and *β*-actin (*n* = 4/group) (b). For western blotting results, a representative blot is shown from four experiments that yielded similar results. Hs27 human dermal fibroblasts were cultured in DMEM until reaching 85% confluence. Cultured human fibroblasts were pretreated with MHY498 for 2 h and exposed to UVB (30 mJ/cm^2^). Type 1 procollagen concentration was measured using a commercially available ELISA kit (*n* = 5/group) (c). Data are represented as mean ± SEM. ^##^ *P* < 0.01 compared to a control group without UVB exposure and ^*∗∗∗*^ *P* < 0.001 compared to the group with UVB exposure.

MMPs are responsible for ROS-induced collagen fiber degradation and inhibition of collagen synthesis [21]. To examine a molecular signaling pathway responsible for collagen recovery by MHY498 after UVB exposure, we measured protein levels of MMP family by western blotting using the dorsal skin homogenate of the hairless mice. UVB exposure increased protein levels of MMP1, MMP9, MMP12, and MMP13, but MHY498 treatment reduced them (Figure 4(a)). In parallel with this, MHY498 treatment recovered the UVB-induced decrease in type 1 and type 3 procollagen protein levels in the skin (Figure 4(b)), indicating that the protective effect of collagen fiber by MHY498 is mediated at least partially through reducing MMP protein levels.

Collagen precursors are produced by fibroblasts in skin and play an important role in maintaining collagen structure [22]. To examine whether MHY498 has protective effect on a collagen precursor in fibroblasts after UVB exposure, we measured intracellular type 1 procollagen (a major collagen precursor in skin) concentration in human dermal fibroblasts cultured primarily from human fetal skin [23] using an ELISA kit. UVB exposure significantly decreased type 1 procollagen concentration in the fibroblasts (Figure 4(c)). On the other hand, MHY498 treatment fully recovered UVB-induced reduction in type 1 procollagen concentration to the level comparable to the control group without UVB exposure (Figure 4(c)). These data suggest that MHY498-mediated type 1 procollagen recovery in the fibroblasts at least partially contributes to the MHY498-mediated protection against UVB-induced collagen destruction.

### 3.5. MHY498 Decreases UVB-Induced Oxidative Stress and Related Signaling

**Figure 5:**
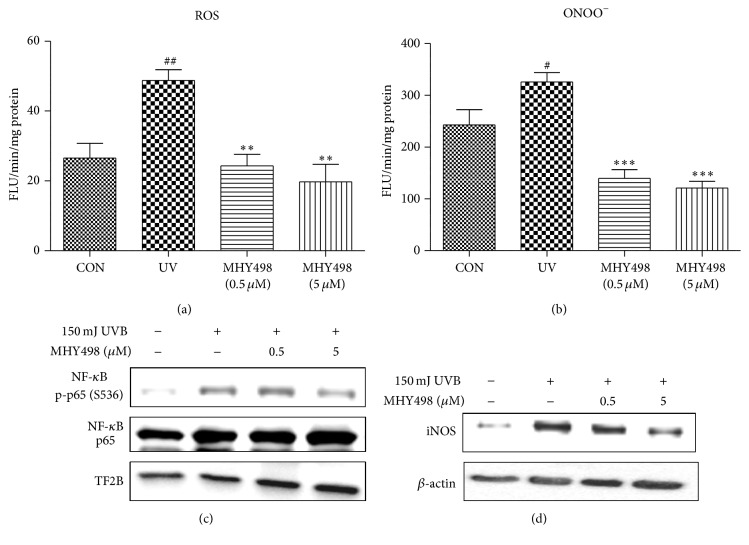
MHY498 reduces UVB-induced oxidative stress and related signaling. MHY498 was pretreated for 3 days. From day 4, 2 h after MHY498 application to the dorsal skin of the hairless mice, UVB was exposed to the mice every other day for 1 h (*n* = 6/group). After 4 weeks, skin samples were excised and ROS (a) and ONOO^−^ (b) generation were measured using DCF-DA and DHR-123 as fluorescent probes (*n* = 6/group). (c) Western blotting images of p-p65 (S536), p65, and TFIIB in the nucleus extracts. (d) Protein levels of NF-*κ*B target gene iNOS in the cytosol extracts. For western blotting results, a representative blot is shown from four experiments that yielded similar results. NF-*κ*B, nuclear factor-*κ*B; TFIIB, transcription factor II B. Each value was expressed as mean ± SEM. ^#^ *P* < 0.05 and ^##^ *P* < 0.01 versus control without UVB exposure; ^*∗∗*^ *P* < 0.01 and ^*∗∗∗*^ *P* < 0.001 versus the UVB-exposed group.

UV exposure induces oxidative stress that is responsible for inducing MMP expression followed by collagen fiber degradation and tyrosinase expression followed by melanogenesis. To examine whether MHY498 decreases oxidative stress after UVB exposure, we measured ROS and ONOO^−^ levels in the skin homogenate of the hairless mice. UVB exposure dramatically increased ROS and ONOO^−^ concentration, whereas MHY498 treatment reduced them to the level comparable to the control group without UVB exposure (Figures 5(a) and 5(b)), suggesting that MHY498 may reduce oxidative stress as an antioxidant. Because UV-induced ROS can activate and translocate NF-*κ*B into the nucleus which is closely associated with skin aging, including collagen degradation and excessive melanogenesis [9, 11, 24], we investigated whether MHY498 inhibits NF-*κ*B activation using skin fibroblasts. It has been reported that phosphorylation of NF-*κ*B p65 on Ser 536 is essential for its capacity to transactivate genes [25]. Therefore, protein levels of p-p65 (S536) and p65 were examined in the nucleus fraction by western blotting. UVB increased the protein levels of p-p65 (S536) in the nucleus, whereas MHY498 reduced it (Figure 5(c)). Consistently, protein level of NF-*κ*B target gene iNOS was increased by UVB treatment but reduced by MHY498 treatment (Figure 5(d)), suggesting that inhibition of NF-*κ*B signaling by MHY498 may contribute to the protective effects on skin pigmentation and collagen destruction against UVB.

Together, in the dermal skin of the HRM-2 hairless mice, MHY498 reduced UVB-induced skin pigmentation by suppressing melanogenesis at least partially through decreases in CREB phosphorylation and MITF translocation to nucleus. In addition, MHY498 inhibited UVB-induced wrinkle formation and collagen destruction, possibly due to inhibiting MMP expression. As a potential mechanism, we assumed that the reduced oxidative stress and NF-*κ*B signaling contribute to the antimelanogenic and antiwrinkle effects of MHY498. Therefore, MHY498 may be applied as a pharmaceutical agent for protecting UV-induced skin damage.

## Supplementary Material

Our supplementary data shows that there are no cytotoxicity in human dermal fibroblasts and B16F10 cells and no DNA damage in the dorsal skin of the hairless mice by MHY498 treatment.

## Figures and Tables

**Table 1:** Treatment scheme for UVB and MHY498.

Day	1	2	3	4	5	6	7	8	9	10	11	12	13	14	15	16	17	18	19	20	21	22	23	24	25	26	27	28

UVB 150 (mJ/cm^2^)				x		x		x		x		x		x		x		x		x		x		x		x	x	x

MHY498	°	°	°	°	°	°	°	°	°	°	°	°	°	°	°	°	°	°	°	°	°	°	°	°	°	°	°	°
