# Supplementary material for: (Z)-5-(2,4-Dihydroxybenzylidene)thiazolidine-2,4-dione Prevents UVB-Induced Melanogenesis and Wrinkle Formation through Suppressing Oxidative Stress in HRM-2 Hairless Mice
Source: Oxid Med Cell Longev. 2016 May 8;2016:2761463. doi: 10.1155/2016/2761463 (PMC4875997; doi:10.1155/2016/2761463)
Supplement: Supplementary file 1 — Our supplementary data shows that there are no cytotoxicity in human dermal fibroblasts and B16F10 cells and no DNA damage in the dorsal skin of the hairless mice by MHY498 treatment. [file 2761463.f1.docx]

**Supplementary figures**

Our supplementary data shows that there are no cytotoxicity in human dermal fibroblasts and B16F10 cells and no DNA damage in the dorsal skin of the hairless mice by MHY498 treatment.

a)

b)

**Supplementary Figure 1. No cytotoxic effects of MHY498 on Hs27 human dermal fibroblasts and B16F10 melanoma cells** MTT assay was performed to investigate cytototoxic effect of MHY498.

**Supplementary Figure 2. MHY498 treatment does not induce DNA damage**

MHY498 was pretreated for 3 days. From day 4, 2h after MHY498 application to the dorsal skin of the hairless mice, UVB was exposed to the mice every other day for 1h. After 4 weeks, the mice were sacrificed and skin samples were homogenated. The nucleus fraction was isolated for western blotting using DNA damage markers. A representative blot is shown from four experiments that yielded similar results
